# A Computer Vision-Based Approach for Tick Identification Using Deep Learning Models

**DOI:** 10.3390/insects13020116

**Published:** 2022-01-22

**Authors:** Chu-Yuan Luo, Patrick Pearson, Guang Xu, Stephen M. Rich

**Affiliations:** Department of Microbiology, University of Massachusetts, Amherst, MA 01003, USA; chuyuan@umass.edu (C.-Y.L.); pbpearson@umass.edu (P.P.); gxu@umass.edu (G.X.)

**Keywords:** medical entomology, ticks, computer vision

## Abstract

**Simple Summary:**

Ticks are ectoparasites of humans, livestock, and wild animals and, as such, they are a nuisance, as well as vectors for disease transmission. Since the risk of tick-borne disease varies with the tick species, tick identification is vitally important in assessing threats. Standard taxonomic approaches are time-consuming and require skilled microscopy. Computer vision may provide a tenable solution to this problem. The emerging field of computer vision has many practical applications already, such as medical image analyses, facial recognition, and object detection. This tool may also help with the identification of ticks. To train a computer vision model, a substantial number of images are required. In the present study, tick images were obtained from a tick passive surveillance program that receives ticks from public individuals, partnering agencies, or veterinary clinics. We developed a computer vision method to identify common tick species and our results indicate that this tool could provide accurate, affordable, and real-time solutions for discriminating tick species. It provides an alternative to the present tick identification strategies.

**Abstract:**

A wide range of pathogens, such as bacteria, viruses, and parasites can be transmitted by ticks and can cause diseases, such as Lyme disease, anaplasmosis, or Rocky Mountain spotted fever. Landscape and climate changes are driving the geographic range expansion of important tick species. The morphological identification of ticks is critical for the assessment of disease risk; however, this process is time-consuming, costly, and requires qualified taxonomic specialists. To address this issue, we constructed a tick identification tool that can differentiate the most encountered human-biting ticks, *Amblyomma americanum*, *Dermacentor variabilis*, and *Ixodes scapularis,* by implementing artificial intelligence methods with deep learning algorithms. Many convolutional neural network (CNN) models (such as VGG, ResNet, or Inception) have been used for image recognition purposes but it is still a very limited application in the use of tick identification. Here, we describe the modified CNN-based models which were trained using a large-scale molecularly verified dataset to identify tick species. The best CNN model achieved a 99.5% accuracy on the test set. These results demonstrate that a computer vision system is a potential alternative tool to help in prescreening ticks for identification, an earlier diagnosis of disease risk, and, as such, could be a valuable resource for health professionals.

## 1. Introduction

Ticks are obligate blood-sucking ectoparasites and are considered second only to mosquitoes as vectors of human disease. Ticks are notorious for their ability to transmit a wide variety of pathogens to humans, including viruses, bacteria, and protozoa. Tick-borne diseases (TBDs) have rapidly become a serious and growing threat to public health in the USA. A total of 649,628 cases of six TBDs were reported to the CDC during 2004–2019 [1]. Moreover, these diseases are often difficult to diagnose or go unreported, so these figures are likely to be an underestimate [2]. Diagnostic approaches rely, in part, on patient symptoms after a confirmed, or suspected, tick bite. Since different tick species are associated with different TBDs, species identification is an essential component of the diagnosis [3]. The TickSpotters program at the university of Rhode Island, for example, provides an online, photograph-based tick identification service [4]. The strategies to determine tick species are either their morphological identification using a taxonomic key [5,6], or a molecular marker analysis using tools such as real-time quantitative polymerase chain reactions (qPCR) [7]. Both approaches, however, require trained experts, specialized equipment, and time to mail or process the tick samples, resulting in a lag to timely treatment. In many instances, the tick identity is unknown and may lead to unnecessary antibiotic administration.

With the development of automated image analysis technology, artificial intelligence has been shown to be a promising solution for various challenges that require specialized and labor intensive image analyses, including medical imaging (X-ray [8], CT scan [9], fMRI [10]), cell image classification [11,12], the monitoring of insects [13], and insect classification [14,15,16]. There are many machine learning algorithms available in the computer vision field. Among them, deep learning algorithms tend to show substantially higher accuracy when the sample size is relatively large [17] and it also achieves a greater accuracy in image classification compared to traditional computer vision techniques [18]. Deep neural networks extract important features from the data automatically without any human supervision, and in some cases, it has been shown to be proficient in classifying images and surpassing human-level performance [19,20]. Unsurprisingly, a recent review analyzed 69 image-based insect identification studies published between 2010–2020 and showed a trend towards applying deep learning techniques [21]. Thus, computer vision and deep learning algorithms could be a powerful tool to replace the current tick identification methods. To date, there have been two studies that have applied computer vision in tick species identification [22,23]. The first study focused on *Ixodes scapularis* (the blacklegged tick) [22] while the second study compared slightly less than 2000 images from four tick species that were captured in the state of Indiana, USA [23]. To improve on this, we sought to develop computer vision algorithms to discern the three major human-biting ticks of North America: *Amblyomma americanum* (lone star tick), *Dermacentor variabilis* (American dog tick), and *Ixodes scapularis*. 

**Figure 1:**
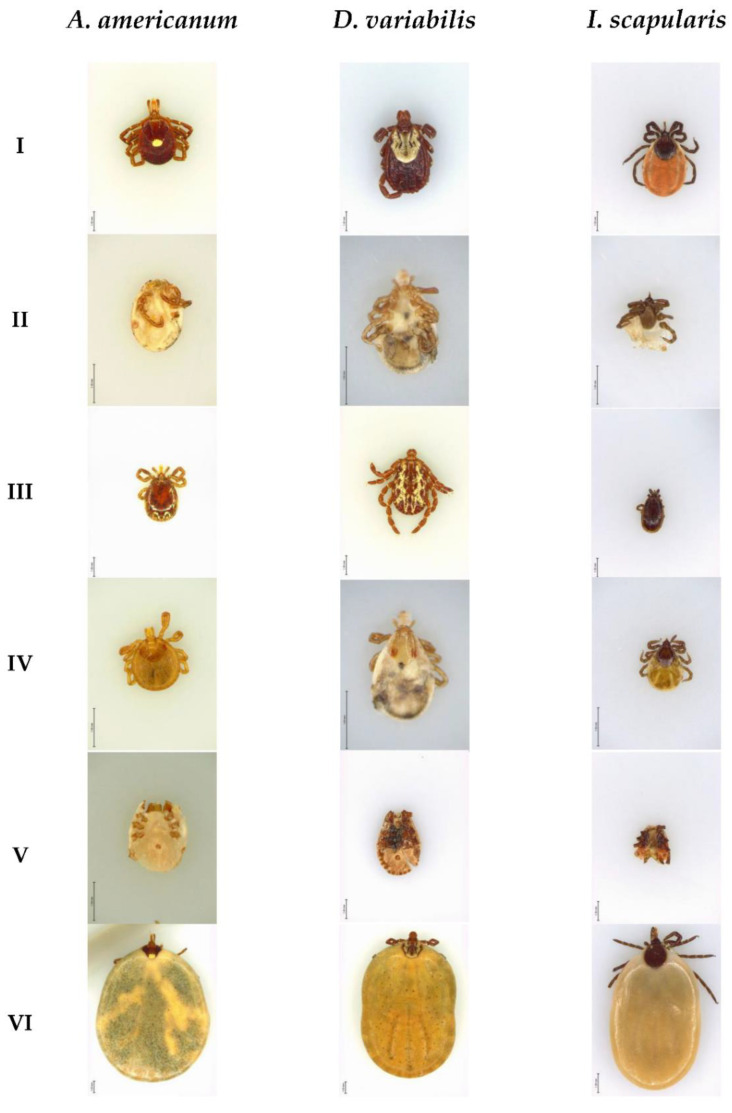
Tick species from the dataset. Inter-species similarity (rows) shows similar traits between different species (*A. americanum*, *D. variabilis*, and *I. scapularis*) and intra-species variability (columns) shows differences such as size, color, and developmental stages within the same species. Row I shows adult female ticks; row II shows ventral view of nymph ticks; row III shows male ticks at adult stage; row IV shows dorsal view of nymph ticks; row V shows ticks with missing body parts; and row VI shows engorged adult ticks. Scale bar corresponds to 1 mm.

The categorization of tick species has two main challenges: different tick species can share morphological similarities (high inter-species similarity) and tick samples from the same species can have significant differences (high intra-species variability). Furthermore, ticks undergo a life cycle accompanied by dramatic morphological changes. For instance, female ticks can increase their weight up to a hundred-fold [24] after blood-feeding and larval ticks only have six legs, whereas nymph and adult ticks possess eight legs. The scutum (a hard chitinous covering) on the nymph and female tick covers roughly half of the dorsal anterior, while the scutum on the male tick occupies the entire dorsal surface. Moreover, the pattern on the scutum changes drastically between different sex and developmental stages. The images in Figure 1 show that ticks within the same species have different developmental stages (nymph, adult), sex (male, female), feeding status (flat, partially fed, engorged) and, therefore, cause a high variance in their visual appearance as well as a high similarity across different tick species. To train a deep learning model, we used a novel large-scale tick dataset, which consists of 12,000 high-resolution micrographs collected from a passive surveillance system. In this dataset, all tick species were molecularly confirmed by a species-specific TaqMan PCR assay, which prevents the human error that may occur in visually identified methods [22,23]. With the ground truth label, this large tick image dataset was applied to several well-known CNN models, such as VGG [25], ResNet [26], Inception [27], MobileNet [28], and DenseNet [29]. Our results showed that a 99.5% classification accuracy can be achieved by applying transfer learning with pre-trained deep convolutional neural networks. We expect that our results will motivate further study on the automatic image-based classification of tick species for the timely detection of potential tick-borne diseases.

## 2. Materials and Methods

### 2.1. Data Sources

**Figure 2:**
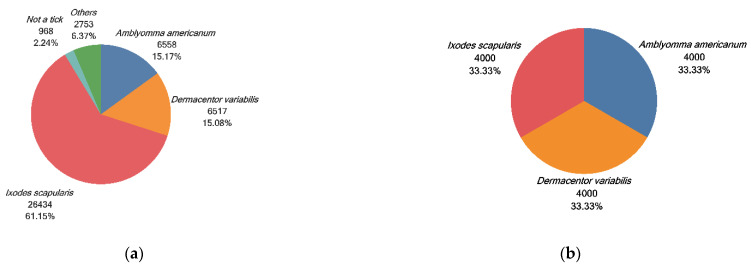
(**a**) Proportion of the tick species to the overall submission; (**b**) proportion of the tick species used in the training process.

Tick samples that were analyzed in the present study were submitted to a passive surveillance tick identification and pathogen testing program (TickReport) from January 2018 through to December 2020 at the Laboratory of Medical Zoology, University of Massachusetts, Amherst. A total of 43,230 tick specimens across the United States were received and 91.4% of ticks belonged to the three major tick species, as shown in Figure 2a.

All images were captured using a Leica S9i stereo microscope and two high-resolution micrographs (3648 × 2736 pixels), of the dorsal and ventral surfaces, were taken for each tick with a white background. Each image captures a single tick. To prevent human error, the tick species identification was first determined by morphological characterization and then was confirmed by a species-specific TaqMan PCR assay [30]. Because of the computing capability, 12,000 tick images were divided into three groups (*A. americanum*, *D. variabilis*, and *I. scapularis*) and four thousand images were randomly selected for each group, as shown in Figure 2b. This tick image dataset covers different developmental stages (larva, nymph, adult), sex (male, female, unknown), feeding status (flat, partially fed, engorged, replete), and host (human, dog, cat, others). In the experiment, ninety percent (10,800/12,000) of the dataset was used for training and validation, and the other ten percent (1200/12,000) was used for testing purposes. We split our dataset into subsets using stratified random sampling, ensuring that the frequency distribution of the outcome was the same in all subsets to ensure the dataset was balanced. Image augmentation has been implemented to increase the diversity and the number of training images [31]. Here, the images were augmented by randomly rotating up to 20 degrees and zooming in on the data with an up to 0.2-scale increase. To minimize the likelihood of the model overfitting and to minimize selection bias, the images in the test dataset were never seen by the neural network model during the training and validation phases.

### 2.2. DCNN Model Architectures

The deep learning model requires a sufficient dataset to achieve a reliable result, and, in some cases, it may be challenging to collect enough data for training an effective model. Due to the limitations on the number of images, the large deep convolutional neural network (DCNN) models may suffer from overfitting in the training process, resulting in a model that does not generalize well from the training dataset. A popular and practical approach to address this issue is using transfer learning [32], which reuses formerly learned knowledge on related tasks and it is a widely utilized method in the computer vision field [33]. Considering the computing resources and time, we applied five well-known pre-trained models along with their weights (VGG16, ResNet50, InceptionV3, DenseNet121, and MobileNetV2) as our transfer learning architectures. All five models have been previously trained on the ImageNet dataset (including 1000 categories with 1.2 million images) [34].

VGG16 architecture was invented by the Visual Geometry Group of Oxford University [25] and won the 2014 ImageNet Large Scale Visual Recognition Challenge (ILSVRC) task for object localization [35]. This model contains a stack of 13 convolutional layers with 3 fully connected layers and represents a classical deep learning model with roughly 138 million parameters. In contrast, ResNet50 architecture is a residual network with skip connections [26] and represents a large deep learning model. Although ResNet50 is 50 layers deep, it has only 25.6 million parameters. Inception-V3 architecture is the 3rd version of GoogLeNet [36] and has 23 million parameters that are 48 layers deep. The memory requirement and computational cost is much lower than VGG16. DenseNet121 is a densely connected convolutional network architecture which is state-of-the-art, according to the classification results from the ImageNet validation dataset [29]. The size of the parameters was significantly reduced down to 8 million and its design also improves its computational efficiency. Finally, MobileNetV2 architecture was also developed by Google Inc. and is focused on the mobile computer vision application, which has relatively light computational requirements [37]. It is designed based on the prior version, MobileNetV1 [28], using depthwise separable convolution as its basic unit.

All the pre-trained models we use, by default, have 1000 different classes at the output layer. We replaced the fully connected layer of the original model with our own fully connected layer that outputs 3-unit tensors, using the Softmax activation function, to classify the image into its corresponding class (*A. americanum*, *D. variabilis*, and *I. scapularis)*. Then, we trained our models with the Adam optimizer [38] using a batch size of 32 and an initial learning rate of 1 × 10^−5^, to minimize the categorical cross-entropy loss in all the training processes. TensorFlow [39] provided a python application programming interface with tutorials to retrain our models with transfer learning. At this stage, our work does not aim to classify other tick species and non-tick samples.

### 2.3. The Hardware and Software Environment

All the experiments were conducted in Keras and TensorFlow deep learning frameworks. A single workstation PC was employed in the entire process of training, validating, and testing the deep learning models described herein. All the captured tick images were resized to 224 × 224 pixels to reduce the memory usage in the procedures. To avoid over-fitting, a simple data augmentation was used that has been proven to enhance the accuracy of classification problems [31]. The hardware applied included a LENOVO ThinkStation P720 Workstation with 80 GB RAM and an NVIDIA GeForce GTX 1080. The software used to train our model was based on Python 3.8.5, Keras 2.4.0, and TensorFlow 2.4.1. More information about the configuration of hardware and software environments can be found in Table 1.

### 2.4. Training Process

**Figure 3:**
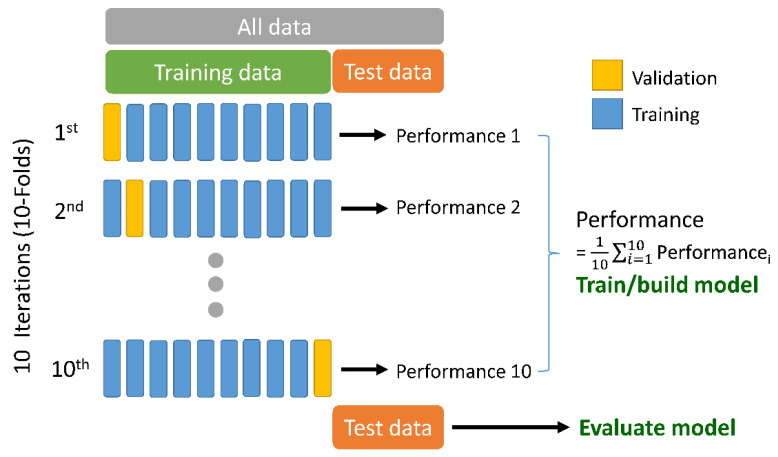
Schematic overview of the 10-fold cross-validation and the excluded test dataset.

To verify the experimental results, a 10-fold cross-validation technique was adopted to evaluate each model’s performance. The tick dataset was divided into two parts: a training dataset and a test dataset. For all the data, 90% was used for training and the validation of the process and the other 10% of the data was reserved for testing. The test dataset was used to evaluate the model. The training dataset was divided into ten subsets containing an equal number of ticks of each species. The training process was carried out 10 times, where each time one part was excluded as validation data, and the remaining dataset was used to train the model. Figure 3 shows the segmentation applied to the dataset.

## 3. Results

### 3.1. Performance Metrics

After training, the accuracy from all subsets was used to obtain a mean accuracy and loss. The accuracy was calculated as the ratio between the number of correct classifications and the total number of classifications. The test accuracy at the end of 20 epochs for each model showed how well the models performed on data they did not previously see (Table 2). The Inception-V3 model achieved the best results with an accuracy of 99.5% and a loss of 0.01. The ResNet50, VGG16, DenseNet121, and MobileNetV2 models achieved accuracies of 99.42%, 99.37%, 99.2%, and 98.73%, respectively. Overall, the results indicate that all five architectures we applied in this experiment performed exceptionally well.

### 3.2. Classification Results

**Figure 4:**
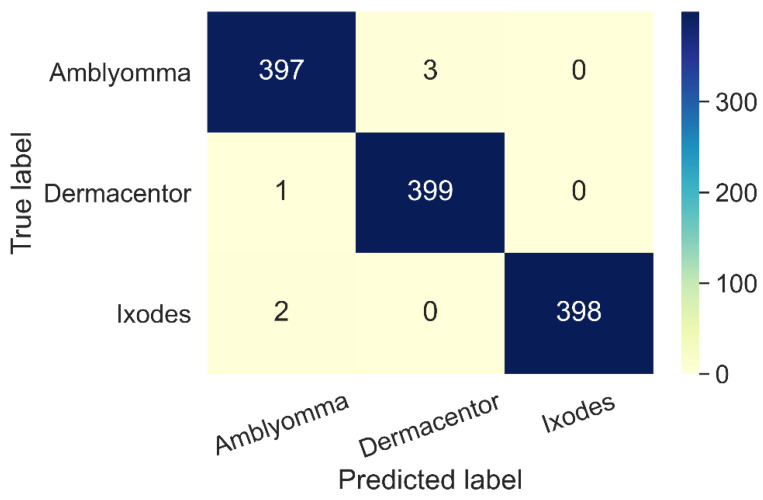
Example results of the confusion matrices from Inception-V3 architecture.

The performance of our best model, InceptionV3, was also evaluated by a confusion matrix (N = 400 images per group) as illustrated in Figure 4. In this experiment, three *Amblyomma* ticks were misidentified as *Dermacentor,* one *Dermacentor* tick was misidentified as *Amblyomma,* and two *Ixodes* ticks were misidentified as *Amblyomma.* Overall, out of 1200 tick images in the test set, only 6 samples were incorrectly identified.

**Figure 5:**
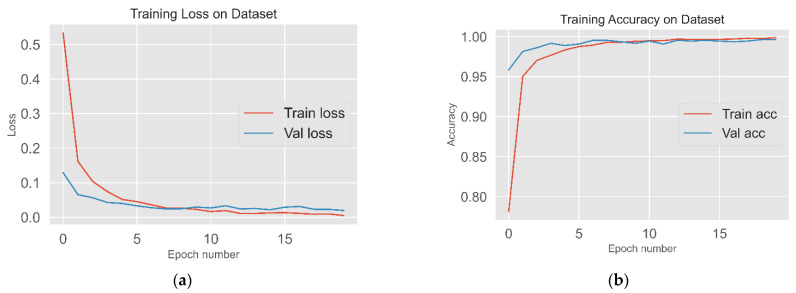
(**a**) Visualizing the loss reduction for the last fold cross-validation training process; (**b**) visualizing the accuracy for the last fold cross-validation training process. Train = training, Val = validation, acc = accuracy.

The loss reduction and accuracy during the training process is shown in Figure 5. The loss in the training and validation sets decreased substantially in the first 5 epochs and the loss tended to stabilize even after the number of epochs increased, indicating that the model has converged. The accuracy of the training and validation sets increased greatly in the first few epochs and stabilized at about 99%, indicating the model training was completed.

## 4. Discussion

The DCNN models are a promising solution in computer vision applications, but they require considerable data for training. The present study adopted transfer learning in addition to the data augmentation methods, such as zooming and rotation, increasing the data size and improving the model generalization ability. The weight parameters of the pre-trained DCNN models were transferred to the new models and then the models were further modified to suit the tick classification tasks. This technique has been heavily used in medical image analyses [40], remote sensing [41], and many computer vision-related fields. To apply the accurate ground truth labels in the training process, our specimens were received through a passive surveillance tick identification and pathogen testing program, and the tick species identification was confirmed by a species-specific TaqMan real-time PCR. The molecular assays ruled out the possibility of misidentification caused by human error or damaged body parts, which are critical for morphological identification.

To our knowledge, only two prior research studies applied computer vision in tick classification. One study used a DCNN model with attention transfer and label smoothing regularization techniques to differentiate *Ixodes scapularis* vs. other ticks and reported an accuracy of 92% [22]. The other study used ResNet50 and custom-built models to predict four tick species and reported an accuracy of 75% and 80%, respectively [23]. Our study focuses on identifying the three most commonly encountered tick species, which includes *Ixodes scapularis*, and the best accuracy achieved was 99.5%. The ResNet50 model has better accuracy in this study compared to the previous report (99.42% vs. 75%). This improvement may be due to providing close to six times more data to the model with photos captured in a standardized setting. Compared to the classical deep learning model VGG16, ResNet50 has less than half the number of parameters but reaches slightly higher accuracy values. The automated tick identification tool developed in this work using the Inception-V3 model obtained the best results, even though the computational cost and memory requirements are much lower than VGG16 and ResNet50. DenseNet121 architecture has fewer parameters, but the accuracy and loss are very similar to VGG16. Lastly, MobileNetV2 has relatively light computational requirements and it still achieves considerable accuracy.

Currently, our work only focused on automated tick identification in a laboratory setting and has achieved noteworthy results. Future model developments may aim towards lighter models which can be deployed on a mobile application for general use. Combining our automated tick identification tool and the long-term passive surveillance data, users can acquire real-time tick species information, as well as the risk of exposure to tick-borne diseases, after uploading tick photos and the location of the specimens. The number of image labels could also be increased to identify more tick characteristics (e.g., developmental stages, sex, and feeding status), which are important factors for risk assessment. A photograph-based tick surveillance program conducted by entomology experts has been shown as a valid method for risk assessment and monitoring among commonly encountered ticks [42]. Our computer vision models could also be integrated into this type of surveillance, without a need for human identification, to provide accurate and timely information for tick control, prevention, and to further combat the rising cases of tick-borne diseases. As tick-borne diseases continue to have an enormous impact, we hope our approach would accelerate the tick identification process and facilitate the early detection of potential tick-borne diseases.

## 5. Conclusions

Deep learning has demonstrated exceptional performance in many computer vision tasks [43] and it also has been applied to image based animal species identification in recent studies [44]. To improve the labor-intensive and time-consuming tick identification process, five pre-trained deep learning models were used to predict tick species and the results of the models were compared. Data augmentation and transfer learning methods were applied during the training phase and the images in the test dataset were never seen by the neural network in the training process. All the pre-trained DCNN models with transfer learning were successful in providing over a 98% recognition rate, indicating that DCNN-based classification approaches are effective for tick species identification.

## Figures and Tables

**Table 1:** Hardware and software environment.

Configuration Item	Value
Type and specification	LENOVO ThinkStation P720 Workstation
CPU	Intel Xeon Silver 4110 2.10 GHz
GPU	NVIDIA GeForce GTX 1080
Memory	80 GB
Hard disk	1 TB
Operating system Image acquisition device	Microsoft Windows 10 Pro Leica S9i stereo microscope
Programming language	Python 3.8.5
Deep learning framework	Tensorflow 2.4.1

**Table 2:** Comparison of different deep learning architectures.

Architectures	Number of Parameters	Accuracy (SD)	Loss
VGG16	138 M	99.37% (±0.29)	0.02
ResNet50	25.6 M	99.42% (±0.17)	0.03
InceptionV3	23.8 M	99.5% (±0.15)	0.01
DenseNet121	8 M	99.2% (±0.29)	0.03
MobileNetV2	3.5 M	98.73% (±0.37)	0.04

## Data Availability

The data presented in this study are available on request from the corresponding author.
